# Analysis of Mechanical Property Degradation of Outdoor Weather-Exposed Polymers

**DOI:** 10.3390/polym14020357

**Published:** 2022-01-17

**Authors:** Sunwoo Kim, Youngmin Lee, Changhwan Kim, Sunwoong Choi

**Affiliations:** 1 Department of Polymer Science and Engineering, Hannam University, Daejeon 34054, Korea; swkpado@krict.re.kr; 2 Chemical Materials Solutions Center, Korea Research Institute of Chemical Technology, Daejeon 34114, Korea; 3 Advanced Materials R&D, LG Chem Ltd., Daejeon 34122, Korea; leeyoungmin@lgchem.com; 4 Climate & Environmental Real-Scale Testing Center, Korea Conformity Laboratories, Jincheon-gun 27872, Korea; kch@kcl.re.kr

**Keywords:** outdoor weathering degradation, mechanical property degradation, performance prediction, fracture strain retention ratio, logistic regression analysis, finite element method

## Abstract

It is well known that many polymers are prone to outdoor weathering degradation. Therefore, to ensure the safety and integrity of the structural parts and components made from polymers for outdoor use, their weather-affected mechanical behavior needs to be better understood. In this study, the critical mechanical property for degradation was identified and modeled into a usable format for use in the virtual analysis. To achieve this, an extensive 4-year outdoor weathering test was carried out on polycarbonate (PC), polypropylene (PP), polybutylene terephthalate (PBT), and high-density polyethylene (HDPE) polymers up to a total UV irradiation of 1020 MJ/m^2^ at a 315~400 nm wavelength. In addition, tensile tests were performed by collecting five specimens for each material at every 60 MJ/m^2^ interval. With the identification of fracture strain retention as the key performance index for mechanical property degradation, a fracture strain retention function was developed using logistic regression analysis for each polymer. In addition, a method for using fracture strain retention function to establish a mechanical property degradation dataset was proposed and successfully tested by performing weathering FE analysis on the virtual automotive collision behavior of a PC part under intermittent UV irradiation doses. This work showed the potential of using fracture strain retention function to predict the performance of polymeric components undergoing mechanical property degradation upon outdoor weathering.

## 1. Introduction

When polymeric materials are applied to structural parts and components for outdoor use, material degradation due to prolonged weather exposure must be considered in the design to ensure safe and reliable lifetime performance [1,2]. This requires a method to generate appropriate mechanical property degradation data and model it into a format for design analysis in virtual engineering. The long-term outdoor weather-affected mechanical property data of polymers are often not readily available, which are essential for successful design analysis of parts made from polymers. Studies on weathering-induced photooxidative degradation on polymers are extensive [3,4,5,6,7,8,9,10]. Discoloration, loss of gloss, refractive index and mechanical property reduction, and peeling of the surface coatings are among the changes occurring from photooxidation [11,12,13]. The mechanism of photooxidative degradation is well known for many polymers. In particular, molecular weight reduction and crosslinking have been identified as major causes of mechanical property changes [4,5,6,7,8,9].

Although many photooxidative studies were conducted using films, fewer studies exist on using bulk specimens. Films were utilized as their primary intent was to characterize the kinetics of degradative chemical reactions. In contrast, bulk specimens were employed to study the deformation and fracture behavior of polymers exposed to UV irradiation [3,4,5]. In bulk specimens, photooxidative degradation is limited to the exposed thin surface layer thickness [6,7] that became brittle [3,4,5,6,7,8,9]. Although the thickness of the brittle surface layer is negligible compared to the total thickness of the specimen, a drastic reduction in deformation and fracture behavior of the specimen occurred [4,5,6,7,8,9,10]. In terms of the loading mode, the static tensile [4,5,6,7,8,9,10], creep and stress-rupture [14,15,16], fatigue [17,18], and impact [19,20] properties were all affected to a large degree. It was shown that when a pre-notched specimen and a specimen with a thin, brittle surface layer produced under UV light were compared under impact loading, the brittle surface layer significantly lowered the impact fracture energy than the pre-notch in the specimen [20]. This was explained as surface layer cracking is an event that imparts kinetic energy to the crack tip and drives the sharp surface cracks further. At the same time, the pre-notch would first undergo crack tip blunting before the impact fracture and hence caused the difference in fracture energies [20]. With regard to the long-term stress-rupture behavior, a drastic reduction in rupture time observed was attributed to the lowering of the crack initiation energy due to the formation of a thin, brittle surface layer by photooxidation [16]. In static tensile behavior, unlike film specimens, where the modulus of elasticity, tensile yield strength, and fracture strains were all affected, the former two properties remained constant with the bulk specimen. In contrast, the fracture strain was significantly reduced [8,10,16,19,21]. This was attributed to the pre-yield surface cracking and post-yield crack growth in ductile polymers, such as polyethylene [14,15,16,20] and polycarbonate [21,22,23]. The same report further determined that the onset of such significant fracture strain reduction occurred when the carbonyl index value reached 0.1 for polyethylene [14,15,16]. It is clear from previous studies that weather exposure degrades the mechanical properties of bulk plastics and affects their deformation and fracture behavior.

Virtual engineering with finite element (FE) analysis is a crucial step in designing robust plastic components. Since most plastic parts are assembled with other parts, they are constantly subjected to internal and external stresses such as clamping force, contact force, internal and external pressures, and thermal stresses. Therefore, virtual stress analysis using long-term mechanical property data obtained from the weathering test is needed to design against weather-induced premature failures. However, the procurement of long-term mechanical property data is not so straightforward, and not only is this capital intensive and time-consuming, but expertise in weatherability testing is essential. Accelerated weathering tests are often used to obtain information about degradation behavior with weather exposure. However, because not all weathering factors can be reproduced in accelerated laboratory tests, realistic base data sets obtained from actual outdoor tests are essential to validate accelerated test results.

Even after obtaining the necessary mechanical property data, it is another challenge to appropriately tailor the data and utilize it to perform weathering FE analysis. The difficulty arises in creating mechanical property data that account for degradation due to weather exposure. At present, no such FE-based design exists to address the weathering effect. As a result, the part designers often try to hedge design risks due to weathering degradation by relying on their design experience as safety factors to the short-term property data provided by the resin suppliers.

This paper presents a 4-year outdoor weathering exposure study on four selected polymers to procure mechanical property changes. It identifies the most appropriate set of weather-affected mechanical property data and proposes a scheme for transforming them into material data for weathering FE analysis. Finally, a virtual product FE analysis involving an impact analysis of PC double chamber channel undergoing weathering degradation was made by applying the material data.

This work is focused on the mechanical property change and its data modeling for FE analysis of long-term outdoor weathering, as it occurs outdoors. The effect of stabilizers, color, and temperature, as well as changes, such as crystallinity, molecular weight, and cross-linking reactions, and the measurement of relevant chemical changes such as the carbonyl index would be subjects for the future work.

## 2. Materials and Methods

### 2.1. Materials

The materials used in this study were polycarbonate (PC), polypropylene (PP), polybutylene terephthalate (PBT), and high-density polyethylene (HDPE). Table 1 illustrates some of the basic properties of the four polymers received from LG Chem, Ltd. (Seoul, Korea). PC (LUPOY PC1303AH, transparent), an injection-grade resin used for outdoor parts and automobile headlamps. PP (LUPOL HF5157, ivory) contains rubber and talc to make automotive exterior parts (especially bumper fascia). PBT (LUPOX HI1006FA, black) is a blend with PC and is used in electrical components due to flame retardancy and high-impact properties. Finally, HDPE (LUTENE-H ME2500, translucent) is for caps and closures due to enhanced environmental stress crack resistance. PC, PP, and PBT were UV stabilized to prevent premature failures from photooxidation since their application is outdoor specific. However, the types of UV stabilizers were not provided as they are the manufacturer’s proprietary information. HDPE is not UV stabilized as its use is not outdoor specific but requires environmental stress crack resistance. The color of the specimen is known to contribute to the degradation by providing a thermal effect [23]. While light colors tend to cause lower temperature rise, the infrared part of the solar radiation causes a higher temperature rise in the darker color specimen. All specimens were received as injection-molded tensile specimens according to ISO 527 Type 1A for PC and PP and ASTM D638 Type I for PBT and HDPE.

### 2.2. Experimental Methods

#### 2.2.1. Outdoor Weathering

**Figure 1:**
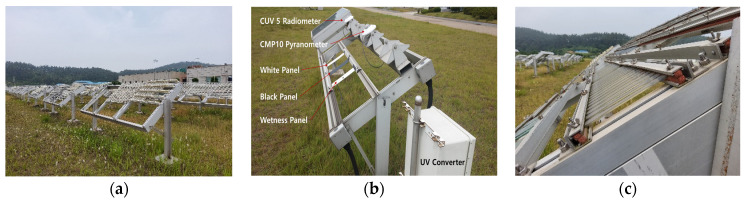
Outdoor weathering test at Seosan: (**a**) overall view; (**b**) integrated station; (**c**) stress-free tensile specimen mounts facing south at a 37° solar angle.

Outdoor weathering tests on four polymers were performed at an outdoor exposure site of Korea Conformity Laboratories (KCL) in Seosan, Korea (Figure 1a), located at the latitude 36°55′ (north) and longitude 126°21′ (east). According to the Köppen–Geiger climate classification, Seosan is a *Cfa* (warm, fully humid hot summer) region [24]. Outdoor weather information was collected daily over a 4-year exposure period (2014–2017) using KCL’s integrated weather station facility. The exposure set up included *Kipp & Zonen* (Delft, The Netherlands) CUV 5 UV radiometer (315~400 nm, sensitivity = 300–500 µV/W/m^2^, non-linearity (0 to 100 W/m^2^) < 1%, operational temperature range = −40 to +80 °C), CMP 10 pyranometer (285~2800 nm, sensitivity = 7 to 14 µV/W/m^2^, Temperature dependence of sensitivity (−10 °C to +40 °C) < 1%, operational temperature range = −40 °C to +80 °C), *Vaisala* (Vantaa, Finland) HMP155 humidity/temperature probe (RH measurement range = 0~100% RH and the accuracy varies with temperature, but has accuracy within ± (1.4 + 0.032 × reading) %RH, and temperature measurement range = −80~+60 °C (−112~+140 °F) and the accuracy varies with temperature, but has ± (0.226–0.0028 × temperature) °C), *Texas Electronics* (Dallas, TX, USA) TE525MM rain gage (Operating temperature range = 0° to 50°C, accuracy = 1.0% up to 50 mm/h (2 in./h)), coated black panel (BP), and wetness panel, as shown in Figure 1b. All specimens were mounted in a patented specimen holder (Figure 1c) that allowed specimens free from thermal expansion and contraction stresses caused by the temperature change.

**Figure 2:**
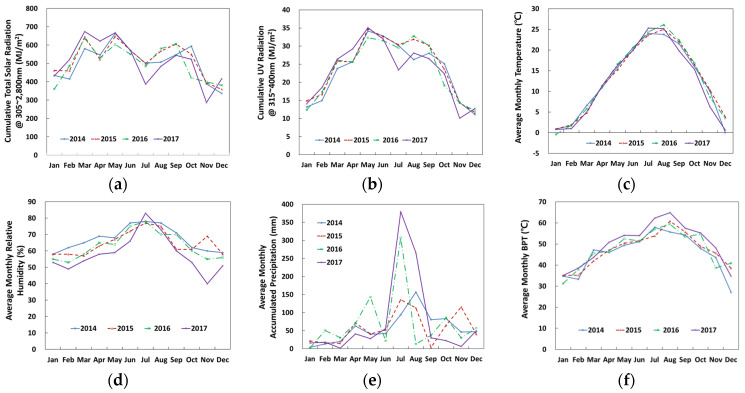
Four-year outdoor weathering information at Seosan, Korea: (**a**) cumulative solar radiation; (**b**) cumulative UV radiation; (**c**) average monthly temperature; (**d**) average monthly relative humidity; (**e**) average monthly cumulative precipitation; and (**f**) average monthly BPT.

Illustrated in Figure 2a–e are monthly cumulative solar radiation (305 nm–2800 nm), cumulative UV irradiation (315 mm–400 nm), average monthly temperature, average monthly relative humidity, and average monthly accumulative precipitation, respectively. In addition, the variation of black panel temperature (BPT) is also given in Figure 2f, which is taken as the surface temperature of the specimen (see Table 2).

With seasonal variation, the monthly cumulative solar radiation was highest between March and May and lowest during November and January (Figure 2a, maximum = 674.0 MJ/m^2^, minimum = 286.8 MJ/m^2^, average = 505.7 MJ/m^2^). Similarly, the cumulative UV light intensity passed through maximum values from June to August and continued to decrease to the lowest values toward December and January (Figure 2b, maximum = 35.1 MJ/m^2^, minimum = 10.1 MJ/m^2^, average = 23.2 MJ/m^2^). Monthly average temperatures were highest from June to August and lowest from December to February (Figure 2c, maximum = 26.1 °C, minimum = 0.4 °C, average = 12.4 °C). The average monthly relative humidity was highest in July and maintained a relative humidity range of 50–75% for 4-year periods (Figure 2e, maximum = 83.0%, minimum = 40.0%, average = 63.3%). Rainfall was concentrated between July and August, with significantly higher precipitation in 2016 and 2017 than in 2014 and 2015 (Figure 2f, maximum = 379.7 mm, minimum = 1.5 mm, average = 64.63 mm). Therefore, a local dip in solar radiation and UV intensity for July was probably due to the concentrated rainfall. Weather variations from 2014 through 2016 remained similar, and in 2017 the change was more pronounced (Figure 2). At the time of planning this study, the annual cumulative UV radiation of the Seosan area was estimated to be 240 MJ/m^2^. However, it was determined to be 283.5 MJ/m^2^.

**Figure 3:**
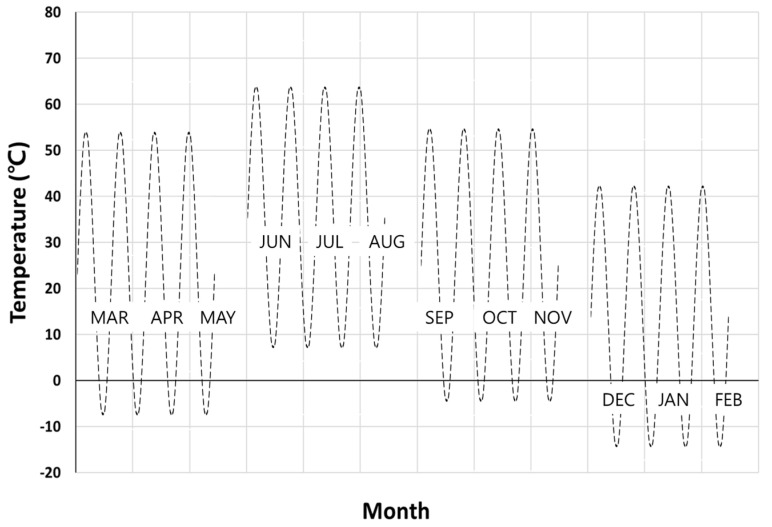
Four-year average quarterly seasonal temperature cycle estimated from the monthly average cycle (temperature varied daily, and the graph is a scheme where daily temperatures are made into a monthly average).

Table 2 shows the 4-year monthly average BPT at Seosan. The changes are significant and were the result of daily, monthly and seasonal climate in Seosan. Based on the BPT change, a daily temperature cycle was produced, and then a quarterly seasonal temperature cycle was created, as shown in Figure 3.

**Figure 4:**
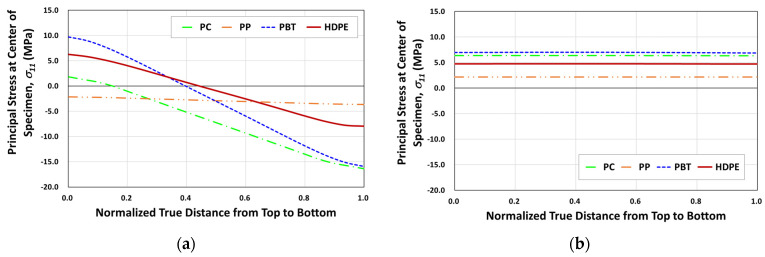
Through thickness variation of principal stress at the center point of the tensile specimen: (**a**) temperature rise (20 °C to 65 °C); (**b**) temperature drop (65 °C to −13 °C).

**Figure 5:**
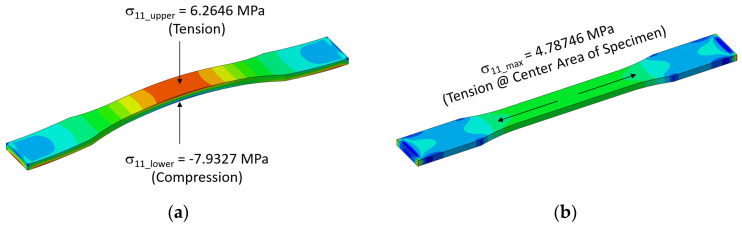
Principle stress ($\sigma_{11})$ contours: (**a**) temperature rise; (**b**) temperature drop.

**Figure 6:**
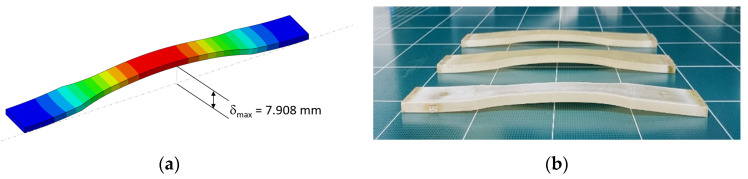
(**a**) Vertical deflection contour at temperature rise, and (**b**) thermal cyclic deformed specimen.

Because of such cyclic climate change, particularly the temperature, thermal cyclic stresses can be imposed on specimens during outdoor exposure if specimens are not properly mounted. For the proper design of the specimen mounting jig, the effect of cyclic temperature variation on the development of thermal cyclic stresses on specimens was first analyzed. Figure 4 shows the principal stress ($\sigma_{11})\;$ development on tensile specimens of four polymers under thermal expansion and contractions from temperature variation when specimens were mounted with two ends fixed. For example, the finite element result has shown that for a temperature rise from 20 °C to 65 °C (seasonal highest BPT), the surface of the outer center portion can attain as high as 6.3 MPa tensile stress for HDPE (Figure 5a and Figure 6a) and the corresponding deflection was almost 8 mm (Figure 6a). Temperature decrease to −13 °C (seasonal lowest BPT) similarly created tensile stress (4.8 MPa) at the center portion of the specimen (Figure 5b), while recovered the center point deflection to zero. As a result of such weather-induced thermal cyclic stresses occurring daily, simultaneous with UV exposure, surface micro-cracks are caused to form [14,15,16,20,22], affecting the tensile test result. Moreover, thermal fatigue and creep can cause permanent deformation in the specimen for longer-exposed specimens, as shown in Figure 6b.

When there is permanent deformation, the tensile data could be affected, for example, the modulus of elasticity and the tensile fracture strain. This is because the modulus of elasticity is measured at a small strain (e.g., ISO 527-2), and if the specimen is permanently deformed, as in Figure 6, the initial strain is taken up in straightening the sample. In addition, the modulus data determined would be lower than the actual expected value. Similarly, the fracture strain can also be reduced depending on the permanent bend due to premature surface microcracking, as explained above.

Therefore, a mounting rack was designed and used to hold tensile specimens (Figure 1c), allowing free movement from expansion and contraction and preventing possible environmental stress fatigue on specimens.

#### 2.2.2. Tensile Testing

For each polymer, 100 injection-molded tensile specimens were outdoor exposed, meaning 400 specimens in total for all four polymers. ISO 527 Type 1A specimens (4 mm thickness) were used for PC and PP, and ASTM D638 Type I specimens (3.2 mm thickness) were used for PBT and HDPE. All specimens were mounted in a test rack that allowed the free movement of the specimens during exposure, as described above (Figure 1c). Five specimens from each polymer were taken at every 60 MJ/m^2^ UV exposure interval up to a cumulative maximum of 1020 MJ/m^2^. In terms of UV radiation dose (*D*) at 315~400 nm, the corresponding exposure time in days in Seosan is given in Table 3 for the total outdoor weather exposure durations. All exposed specimens were conditioned at 23 °C and 50% RH for at least 10 days prior to performing tensile tests. The tensile test on the exposed specimens was conducted in the same environment as the conditioning using a universal testing machine (Zwick Z010). A contact extensometer with a 50 mm gage length was employed. The rate of displacement was 1.0 mm/min up to a strain value of 0.3% and was changed to 50 mm/min until specimen failure. A stress–strain curve, modulus of elasticity, tensile yield strength, and fracture strain were obtained through a tensile test.

## 3. Results and Discussion

### 3.1. Tensile Behavior

**Figure 7:**
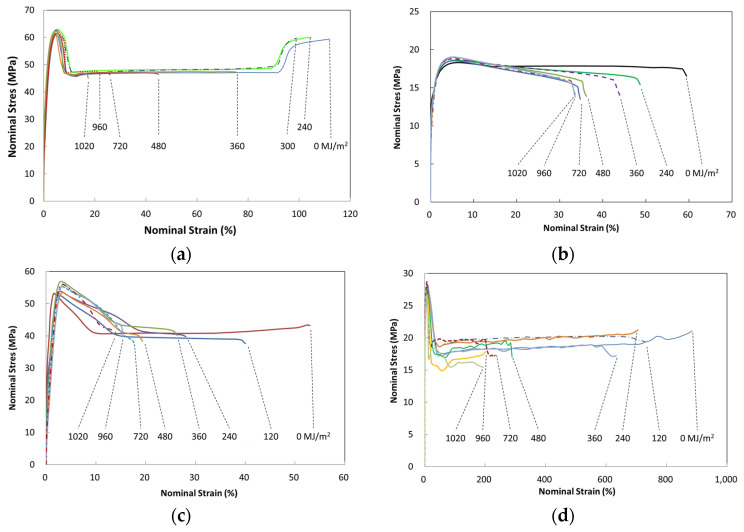
Nominal tensile stress–strain curves of weather-exposed polymers during a 4-year weathering test: (**a**) PC; (**b**) PP; (**c**) PBT; and (**d**) HDPE.

**Figure 8:**
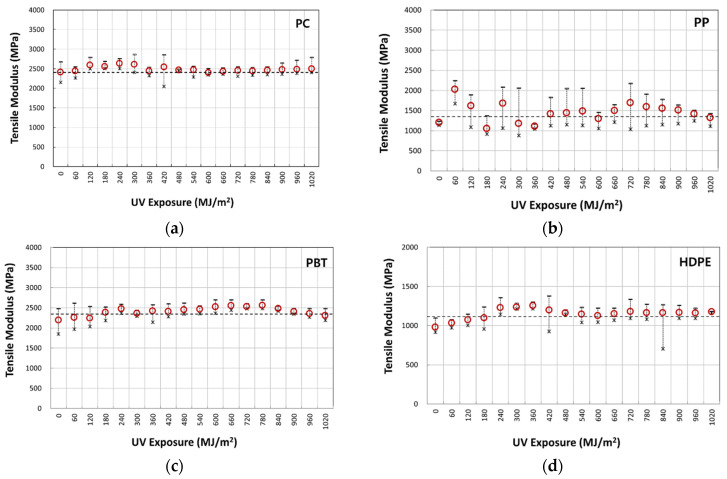
Variation of tensile modulus with UV irradiation dose: (**a**) PC; (**b**) PP; (**c**) PBT; and (**d**) HDPE.

**Figure 9:**
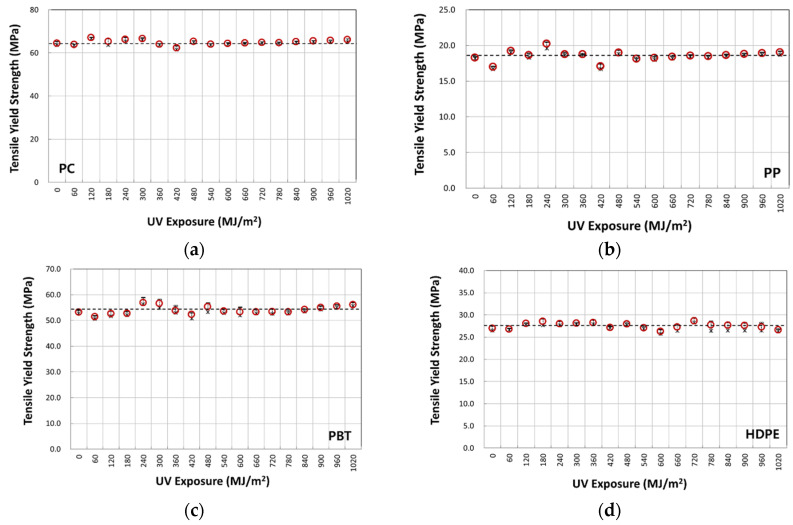
Variation of tensile yield strength with UV irradiation dose: (**a**) PC; (**b**) PP; (**c**) PBT; and (**d**) HDPE.

**Figure 10:**
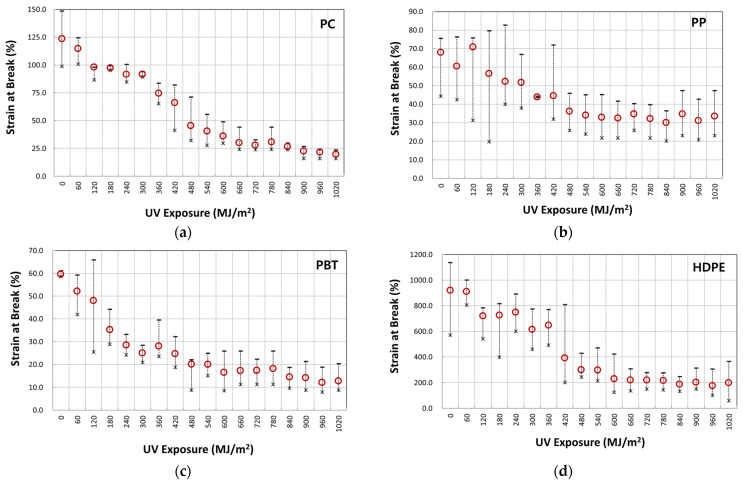
Variation of fracture strain with UV irradiation dose: (**a**) PC; (**b**) PP; (**c**) PBT; and (**d**) HDPE.

Figure 7a–d illustrate typical nominal stress–strain behavior of four-year outdoor exposed specimens for PC, PP, PBT, and HDPE, respectively. The graphs shown were from selected UV dose intervals to illustrate the difference in fracture strain values. The full set of UV dose-affected properties are shown in Figure 8, Figure 9 and Figure 10. Tensile test data indicate that the modulus (Figure 8), tensile-yield strength (Figure 9), yield strain did not vary to a noticeable degree (within standard deviation) even after 4 years of outdoor exposure. Their means values are tabulated in Table 4. On the other hand, significant changes occurred in fracture strain values upon outdoor exposure, as shown in Figure 10.

For example, polymers all show an initial plateau where the fracture strain change is minimal, then a rapid decrease followed by convergence to a minimum limiting value. Fracture strain of PBT decreased from the original 58% to a minimum of 16%, PP from 67% to 31%, PC from 117% to 19%, and HDPE from 897% to 175%. This difference in post necking is due to the different chain and crystal structures of the polymers.

In general, the stress–strain curves of all four polymers exhibit more similar behavior at small strains than at large strains. Similar strain–strain behavior was also obtained in many UV–exposed polymers [4,5,6,7,8,9,10,19,21]. This is expected as the UV degradation is confined to a thin surface layer in the order of hundreds of microns thick, which is much smaller than the total specimen thickness.

The microcrack in a thin surface layer can be produced due to the molecular weight decrease from the photooxidative reaction [14,15,16,22], making the surface to become brittle. Additionally, the chemi-crystallization induced by the molecular weight decrease would further contribute to forming a brittle surface [16]. The same paper also reported that the number of chain scission per chain molecule to cause rapid strain reduction for polyethylene was less than 1.0. This produced a crystallinity increase of about 10%. It should be noted that the crystallinity change was reported to occur more in the summer than in the winter season [25]. However, what seems to be a slight seasonal increase or decrease in the data shown, for example, in Figure 8d for the tensile modulus, would most likely be due to the experimental scatter in the measurement. This is because the crystallinity change only occurred at the very thin surface layer. Hence, if surface microcracking occurs, it would occur before the necking strains. Choi and Broutman reported that even if microcracks are formed on the surface layer prior to the necking strain, behavior in the small strain region is not affected in the bulk specimen due to the thin layer being much thinner (50~200 µm) than the total specimen thickness (4 mm) [14,15,16]. They also reported that above the necking strains, where the neck instability starts to grow, the microcracks would then start to have the effect of crack growth, as the neck instability propagation would decrease the width and the thickness of the specimen. They further went on to report that when the full neck is produced and cold drawing occurs at constant stress, the cross-sectional area of the specimen is at its minimum, which would effectively make the condition equivalent to the crack being longer. That is, since the thickness of the tensile specimen at large strains from cold drawing is at its minimum, the surface cracks have the effect of getting longer compared to the surface crack length at the original specimen thickness. Hence, these surface cracks can grow through the specimen and shorten the cold drawing ability to produce large strains. Therefore, in thicker specimens, whose thin surface layer is degraded by the UV exposure, the properties which are dependent on small strains (modulus of elasticity, tensile yield stress, and yield strain) are not affected, whereas the large strain regions of neck growth and cold drawing are affected significantly. Support of this behavior is given by the studies showing the modulus of elasticity and tensile yield stress of a 200 µm thin polyethylene film increasing significantly with UV exposure times [14,15,16]. It also showed large fracture strain reduction, following the crystallinity increase and molecular weight decrease. In another study, a 6.35 mm thick specimen was tensile tested after UV exposure produced a 150 µm thin brittle surface layer [14]. They have shown that when the specimen thickness is reduced by the layer removal from the unexposed side to a total specimen thickness of 900 µm and thinner, the modulus increased exponentially from the original unexposed value. They attribute this to the relative volume increase of the thin brittle surface layer.

Therefore, from the appearance of the stress–strain curves in Figure 7a–d, for PC, PP, PBT and HDPE, the cold-drawing strains are progressively shortened while diffused necking strains in PP are affected by increasing UV exposure. In Figure 7, the variation of stresses observed in the strain range from the onset of necking to full neck formation is larger for PBT and HDPE than the PC and PP. Furthermore, HDPE shows the most fluctuation (max ± 7.5%) in the strain range between full neck formation and cold drawing to failure, where it occurs at more or less constant stress. These post-necking behaviors become more sensitive to the surface layer cracking and growth. Furthermore, this is most likely due to PBT being an aromatic polyester and HDPE not being UV stabilized, making them more prone to surface layer degradation on UV exposure. In turn, this would also make them more sensitive to specimen preparation and deviations in the tensile test (e.g., HDPE in Figure 7d, the post necking stress–strain curve of 0 MJ/m^2^ and 360 MJ/m^2^ matches, and 120 MJ/m^2^ and 240 MJ/m^2^ are in a match). In addition, PBT is known to become more sensitive to photo-degradation, particularly when moisture is present, as in outdoor weathering [23]. In addition, the color of PBT being black may also have contributed to the increase in the rate of degradation due to the temperature rise upon radiative heat absorption. Another factor may be that with the high ductility of HDPE (large elongation ~1000%), the initial surface microcracks created before necking strain produce different surface crack lengths depending on the amount of UV exposure received. These cracks then would have the effect of growing deeper or shorter into the material from the onset of necking. Therefore, depending on the crack growth effect into the bulk specimen from the point of full neck formation, the larger the growth, the lower the stress–strain curve at those affected strain regions, and therefore appearing as fluctuations in the stress–strain curve observed.

From the above, the fracture strain reduction can be identified as the source of mechanical property degradation occurring during prolonged outdoor weathering. Hence, the generation of material data for weathering FE analysis would involve fracture strain reduction with weather exposure and other mechanical properties maintained at constant values.

### 3.2. Logistic Regression Analysis of Fracture Strain Retention

**Figure 11:**
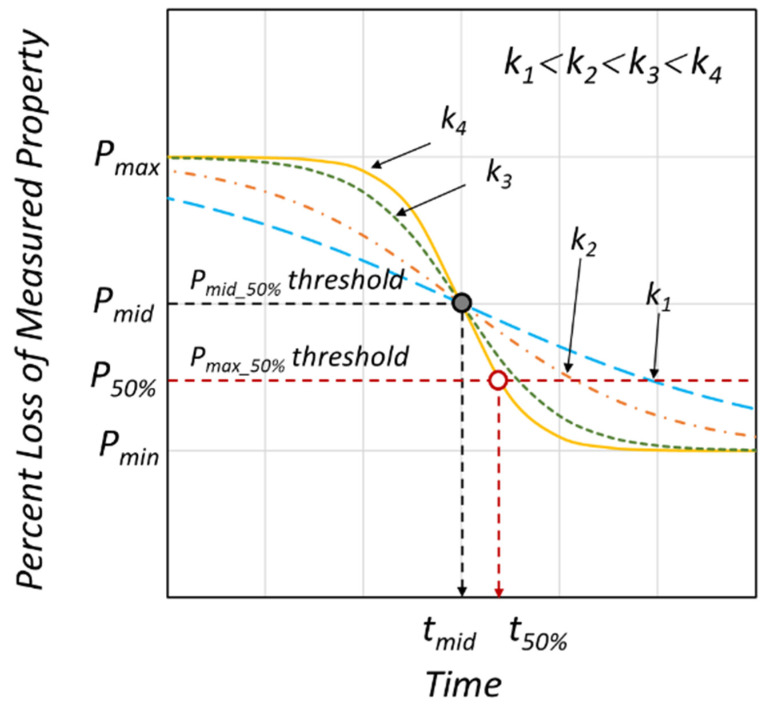
Schematic representation of the inverted S-shaped property retention model.

From observing the fracture strain change on four polymers that had undergone 4 years of weathering (Figure 10), the characteristic behavior can be described by a logistic regression model of the inverted S-shape function, as shown in Figure 11. In this model, the property retention (e.g., fracture strain retention) is maintained for the initial period, followed by an accelerated decrease, and converges to some constant value. Thus, the property retention with time in Figure 11 can be expressed by Equations (1) and (2).

(1) $$P\left(t\right)=P_{min}+\frac{P_{max}-P_{min}}{1+exp^{-k\left(t-t_{0}\right)}}$$

(2) $$t=\frac{1}{k}log_{e}\left(\frac{P\left(t\right)-P_{min}}{P_{max}-P\left(t\right)}\right)+t_{0}$$

where *t, P_min_, P_max_* are the time, minimum, and maximum retention values, respectively; *k* is the logistic growth rate or steepness of the curve, and *t*_0_ is the midpoint of the inverted S-shaped curve (*t_mid_*). For 0.5 > *P_min_* > 0.0, *P_mid_* is the 50% retention value at *t*_50%_ and for *P_min_* = 0.0, the retention value at t_50%_ becomes *P_max_*/2, as calculated by Equations (1) and (2). When *P_min_* > 0.5, *P_min_* is a limiting value below which the weathering exposure does not cause any further reduction in the property. In References [4,5,6,7,8,9,10,19,21], similar to the inverted S-shaped curve shown in Figure 10, the tendency of the fracture strain to decrease with the lapse of weathering time was confirmed.

**Figure 12:**
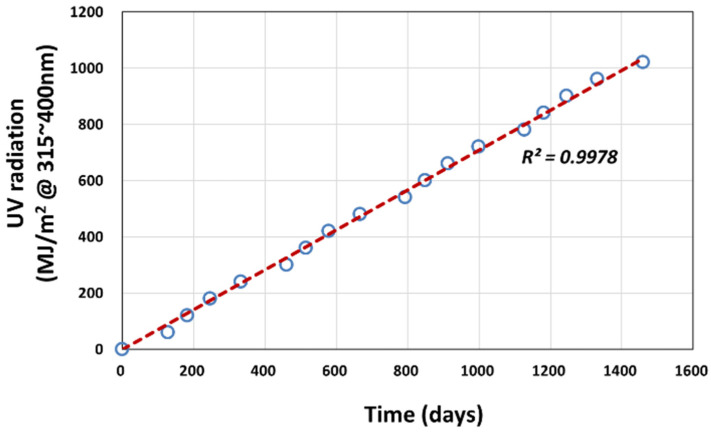
Cumulative UV radiation at 315–400 nm received (*D*) versus outdoor exposure time (*t*) in Seosan.

In the logistic regression analysis, the actual weathering exposure time (*t*) is converted to the cumulative UV (@ 315–400 nm) radiation dose received (*D*) using Equation (3), based on the measurement made at Seosan as shown in Figure 12. This is more plausible as the actual tensile test was conducted on specimens taken at every 60 MJ/m^2^ dose interval.

(3) $$t_{days}=1.4101\times D$$

In terms of UV irradiation dose, *D*, the nominal fracture strain retention ($P_{e_{f}}\left(D\right)=e_{f_{D}}/e_{f_{0}}$) is given as the ratio of fracture strain at specific UV exposure dose ($e_{f_{D}})$ normalized by the fracture strain value of the unexposed specimen ($e_{f_{0}})$ as shown in Equations (4) and (5):

(4) $$P_{e_{f}}\left(D\right)=\frac{e_{f_{D}}}{e_{f_{0}}}=P_{min}+\frac{P_{max}-P_{min}}{1+exp^{\left(k\left(D-D_{0}\right)\right)}},\;\;P_{e_{f}}\left(0\right)=1,\;\;P_{min}\geq 0$$

(5) $$D_{50\%}=\frac{1}{k}log_{e}\left(\frac{P_{max}-0.5}{0.5-P_{min}}\right)+D_{0}$$

Here, *k* is the logistic growth rate or steepness of the curve, *D*_0_ is the midpoint (*D_mid_*) of the inverted S regression model, and *D*_50%_ is the corresponding accumulated UV radiation dose when the fracture strain drops to 50% of the initial unexposed value as shown in Equation (5). Hence, if *P_min_ >* 0, *D*_0_ *≠ D*_50%_ and if *P_min_ =* 0, then *D*_0_ *= D*_50%_. The four parameters of *P_min_, P_max_, k, and D*_0_ in Equation (4) were obtained by implementing the Generalized Reduced Gradient (GRG) nonlinear algorithm in Excel as a *Solver* function [26,27]. The coefficient of determination of fracture strain, *R^2,^* was calculated using the *sum of squares regression* (*SSR*-Equation (6)) and the *total sum of squares* (*SST*-Equation (8)) in Equation (9). The *SST* is equal to *SSR + SSE* as shown in Equation (8), and the *sum of squares of error* (*SSE*-Equation (7)) is that least-squares regression selects the line with the lowest total sum of squared prediction errors.

(6) $$SSR=\sum {\left(\hat{P}-\bar{P}\right)}^{2}$$

(7) $$SSE=\sum {\left(P-\hat{P}\right)}^{2}\;$$

(8) $$SST=SSR+SSE=\sum {\left(P-\bar{P}\right)}^{2}$$

where $\bar{P}$ is the mean of P and $\hat{P}$ is the estimate of P

(9) $$R^{2}=\frac{SSR}{SST}=\frac{SSR}{SSR+SSE}=1-\frac{SSE}{SST}$$

**Figure 13:**
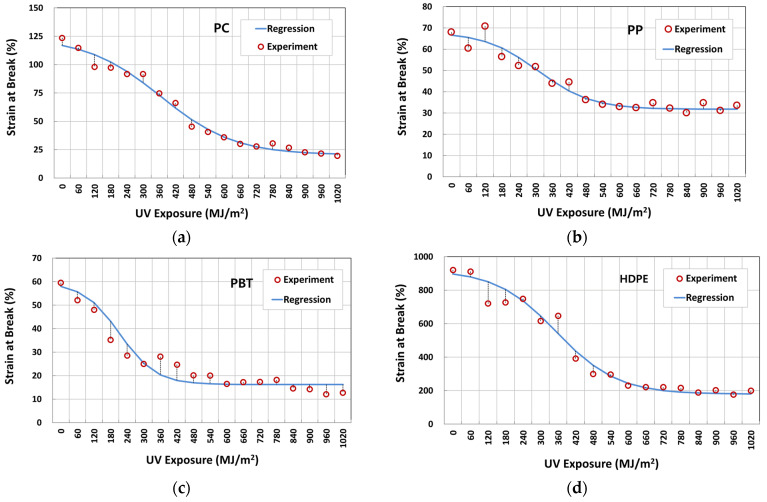
Fracture strain data and logistic regression results of four polymers during the 4-year weathering test: (**a**) PC; (**b**) PP; (**c**) PBT; and (**d**) HDPE.

**Figure 14:**
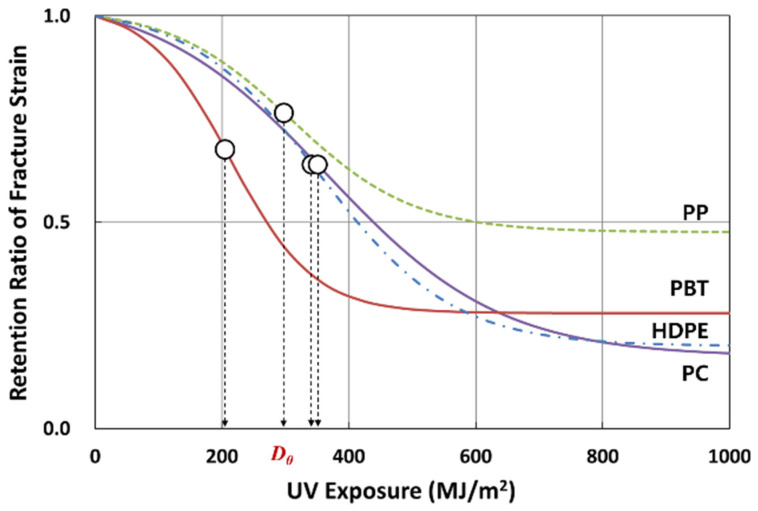
Comparison of fracture strain retention between PC, PP, PBT, and PE during 4-year weathering test. Midpoint positions (*D_0_*, open circles) are shown for each polymer.

The logistic regression analysis results on fracture strain data are given in Table 5. The corresponding regression curves for PC, PP, PBT, and HDPE are presented in Figure 13a–d, respectively. The regression was made to fit through *P_max_* = 1.0 for all polymers. In all cases, the *R*^2^ values were higher than 0.93, indicating that the model can well predict the change in fracture strain in four polymers that underwent 4 years of weathering exposure. Figure 14 (and Table 5) compares the fracture strain retention regression curves of four polymers. In terms of the UV dose required for 50% fracture strain reduction based on the midpoint of the curve, *D*_0_, PBT (213 MJ/m^2^) performed worst, followed by PP (309 MJ/m^2^), HDPE (355 MJ/m^2^), and PC (365 MJ/m^2^). As for the minimum fracture strain retention, PP (0.47) performed best, followed by PBT (0.27), HDPE (0.20), and PC (0.17). *k* values can be also seen to decrease from PBT (0.0153), PP (0.0105), HDPE (0.0096), and PC (0.0073).

### 3.3. Weathering FE Analysis

The results from the logistic regression analysis on the fracture strain retention were further used to develop stress–strain material data for the FE analysis. For this, the true stress–strain curve of the unexposed specimen and the fracture strain retention regression model were utilized. The true stress and true strain were calculated from the nominal-–stress strain data using Equations (10) and (11), respectively.

(10) $$\sigma_{true}=\left\{\begin{array}{c} \sigma_{nom}\left(1+e_{nom}\right),\;\;\;\;\;\;\;\;\;\sigma_{nom}\leq \sigma_{y}\\ \sigma_{y}\left(1+{\left(e_{nom}-e_{y}\right)}^{n}\right),\;\;\;\;\;\sigma_{nom}>\sigma_{y} \end{array}\right.$$

**Figure 15:**
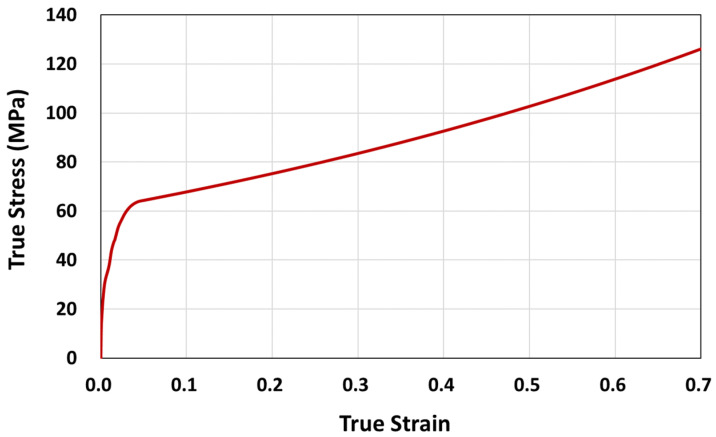
True stress–strain curve for PC.

(11) εtrue=∫l0ldl/l=lnll0=ln1+enom

where, *σ_nom_, e_nom_*, *σ_true_ ε_true_*, *σ_y_*, *ε_n_* are the nominal stress and strain, true stress and strain, tensile yield stress and strain (per ISO 527), and strain exponent, respectively. *l*_0_ is the original distance between gauge marks, *l* is the distance between gauge marks at any time *t*, *dl* is the incremental elongation when the distance between the gauge marks is *l.* The true stress–strain curve of PC constructed using *n =* 1.0 is shown in Figure 15.

The true stress–strain data of polymers were further converted into appropriate elastic-plastic data for FE analysis using ABAQUS [28]. Most of the plasticity models in ABAQUS are incremental theories, and the mechanical strain is decomposed into an elastic part and a plastic (inelastic) part, as shown in Equations (12) and (13).

(12) $$\varepsilon_{total}=\varepsilon_{e}+\varepsilon_{p}$$

(13) $$\varepsilon_{p}=ln\;\left(1+e_{nom}\right)-ln\left(1+\sigma_{y}/E\right),\;\;\;\;e_{nom}\geq e_{y}$$

where, *ε_e_*, *ε_p_*, *E* are true elastic strain, true plastic strain, and tensile modulus, respectively. The independent elastic constants (tensile modulus, *E* = 2489.17 MPa, and Poisson’s ratio = 0.43 [29,30]) were used to describe the elastic part and the first true plastic strain input *ε_p_* = 0, replaces *ε_y_* = 0.022338 which defines the true yield stress, *σ_y_* = 56.2 MPa.

**Figure 16:**
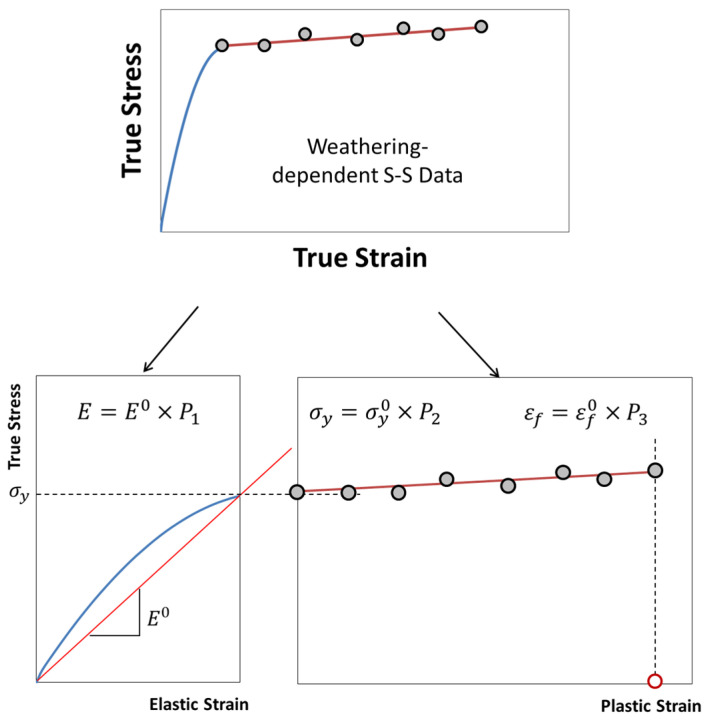
Scheme for generating weathering exposed material model for FE analysis using fracture strain retention value.

For the material data to accommodate the fracture strain reduction with weather exposure, the unexposed mechanical property values of elastic modulus ($E^{0}$), yield strength ($\sigma_{y}^{0})$, failure strain $\left(\varepsilon_{f}^{0}\right)$ and the property retention values (*P_i_*) were utilized. Therefore, the true stress–strain curves maintained the elastic portion of the unexposed polymers while the unexposed fracture strain ($\varepsilon_{f}^{0}$) was multiplied by the fracture strain retention values (*P*_3_) obtained through the logistic regression analysis (Equation (4) and Table 5) in estimating the degradation of the mechanical properties over exposure time (Figure 16). The retention values of elastic modulus (*P*_1_) and the tensile yield strength (*P*_2_) were set to 1.0, as negligible changes were observed during the weathering exposure (Figure 8a and Figure 9a). Based on the nominal fracture strain retention, $P_{e_{f}}$, calculated using Equation (4), the retention for true fracture strain retention can be calculated as in Equation (14):

(14) $$P_{3}=\frac{ln\left(1+P_{e_{f}}\cdot e_{f}\right)}{ln\left(1+e_{f}\right)}$$

where $P_{e_{f}}$ and $e_{f}$ are nominal fracture strain retention and nominal fracture strain, respectively. For the case of films or thin sheets where the modulus and the yield stress are affected with UV exposure, *P*_1_ and *P*_2_ should be determined and applied to estimate the mechanical property degradation.

**Figure 17:**
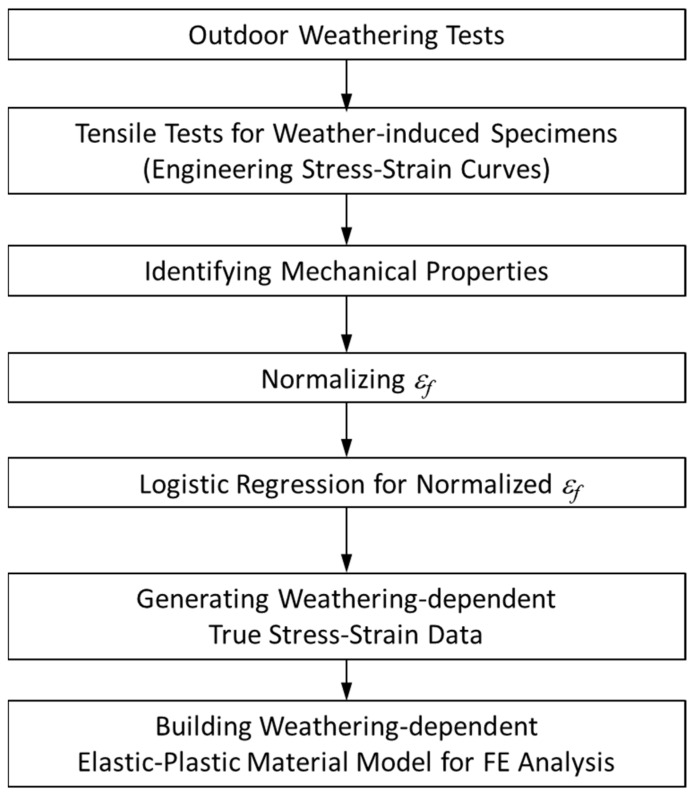
Flowchart of building weather-affected material data for structural FE Analysis.

The material degradation data in ABAQUS was then defined as a function of UV dose, *D*, using user-defined functions (USDFLD of ABAQUS/Standard and VUSDFLD of ABAQUS/Explicit). A flow chart for making weather-affected material data for FE analysis is summarized in Figure 17.

**Figure 18:**
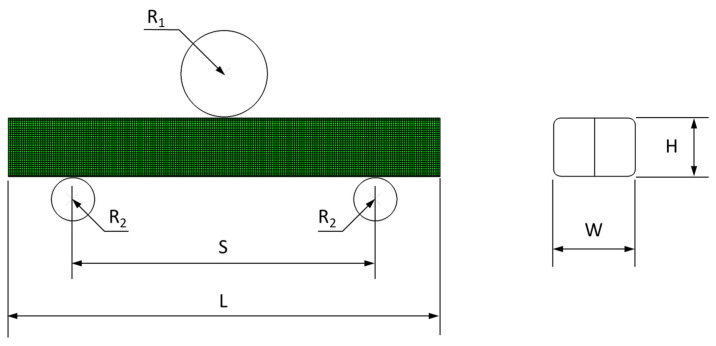
Double-chambered channel three-point impact bending configuration: geometry and finite element mesh. Tup radium (R_1_) = 50 mm; radius of support pin (R_2_) = 20 mm; span (S) = 350 mm; double-chambered channel dimension L × W × H = 500 × 90 × 70 mm^3^.

**Figure 19:**
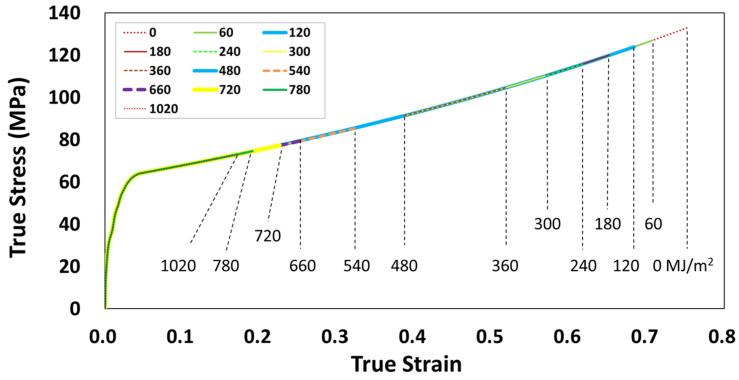
True stress–strain curves of weather-exposed PC used in collision impact simulation.

To demonstrate the implementation of the mechanical property degradation data in a virtual weathering analysis, a discrete time-dependent impact FE analysis was carried out on a part subjected to intermittent weathering exposure. Figure 18 shows the three-point impact bending of an automotive collision absorption component in the form of a double-chambered channel section [31]. For this analysis, the material of the double channel was changed from aluminum to an 8 mm thick PC. PC was selected as they are used in outer automotive structures designed to withstand collisions while exposed outdoors. In addition, the impact was chosen as they can produce large plastic strains and high stresses on structures. According to the flow chart shown in Figure 17, the stress–strain curves of the weather-exposed PC material were generated as shown in Figure 19. These curves were used in this simulation. The three-point bending test configuration consists of two support pins (radius 25 mm) and a central punch (radius 50 mm). The distance between the pins is 350 mm. The tup and the supports are modeled as rigid bodies. The PC double-channel component was modeled with a fine mesh (about 5 mm edge length) using a 4-node shell element (S4R). The friction coefficient at the contact between the rigid tup and the PC component was 0.05. The friction coefficient of 0.15 was used for the contact between PC self parts. The dynamic bending impact tests were conducted using an impact velocity of 2 m/s. ABAQUS/Explicit is used as it is particularly suitable for simulating transient dynamic events as in automotive crashworthiness, where inertia is a variable in calculating dynamic equilibrium. The FE analysis was carried out to compare the difference in the impact behavior of the channel section according to the weathering exposure. As used in the reference [28,31], the same collision condition was utilized for the FE analysis.

**Figure 20:**
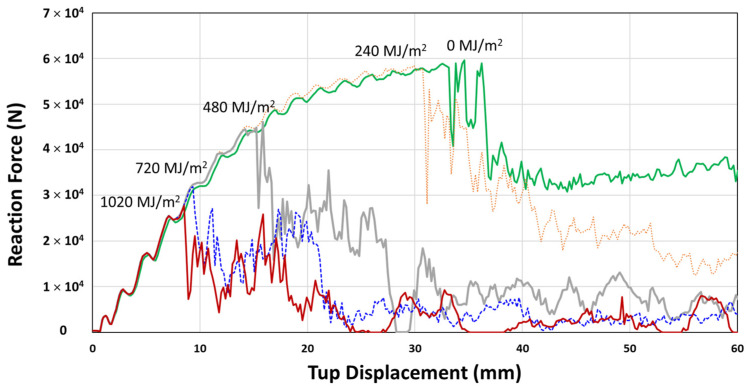
Reaction force versus tup displacement curves of 3-point bending impact analysis for weather-exposed PC channel section.

**Figure 21:**
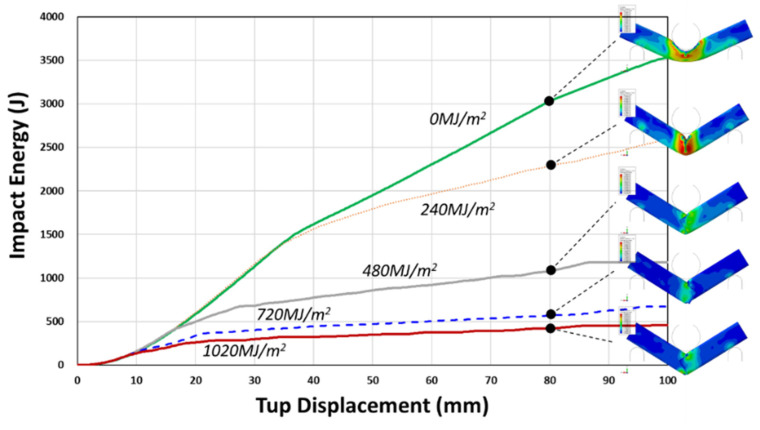
Impact energy versus tup displacement curves of 3-point bending impact analysis for weather-induced PC channel section.

Crashworthiness simulation was performed for 0, 240, 480, 720, and 1020 MJ/m^2^ UV radiation doses. The reaction force and impact energy with impact tup displacement is shown in Figure 20 and Figure 21, respectively. The characteristic sudden drop after the initial reaction force increases to a maximum value is taken as the fracture (crack) initiation point (Figure 20). The fracture initiation point corresponds to that displacement of the moving tup during three-point impact loading. It is seen to decrease with UV exposure from approximately 35 mm, 31 mm, 15 mm, and less than 10 mm for 0, 240, 480, 720, and 1020 MJ/m^2^, respectively. The impact energy at the maximum reaction force (fracture initiation energy) and the total impact energy determined from Figure 20 and Figure 21 are summarized in Table 6.

The fracture initiation energy is seen to decrease by 16.8% at 240 MJ/m^2^ UV radiation dose, and at 480 MJ/m^2^ dose, the initiation energy reduces rapidly by 72%. Finally, at a still higher UV dose of 720 MJ/m^2^ and 1020 MJ/m^2^, the reduction approaches a limiting value of about 90~92%. Similarly, the initial decrease of total impact energy, determined at 60 mm impact tup displacement, is 14.8%, followed by a more rapid decrease by 60.1% at 480 MJ/m^2^ UV dose. However, the rate of total impact energy decrease at higher UV doses, 720 MJ/m^2^ (78.1%) and 1020 MJ/m^2^ (83.7%), were less than the rate determined for the fracture initiation energy. Since the total impact energy is the combination of fracture initiation and propagation energies, this implies that the fracture initiation is more affected than the propagation by the UV exposure (0% (0 MJ/m^2^) → 11.8% (240 MJ/m^2^) → 42% (480 MJ/m^2^) → 59% (720 MJ/m^2^) → 71% (1020 MJ/m^2^)), as summarized in Table 6.

Therefore, the mechanical property degradation data set generated and applied to FE analysis have been shown to perform the prediction of the structural performance of weather-exposed polymeric parts.

**Figure 22:**
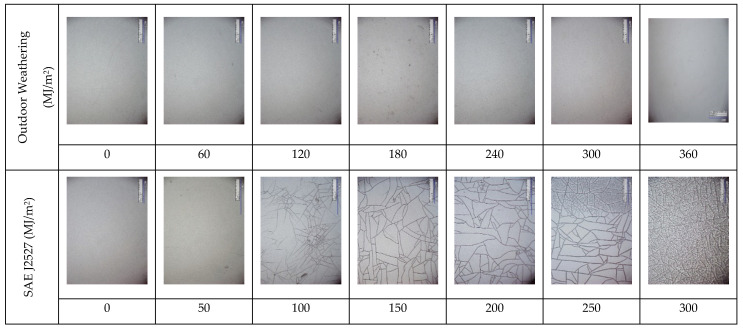
Microscopic images (×350) of weather-exposed surfaces of polycarbonate between natural outdoor weathering at Seosan and accelerated weathering test per SAE J2527.

As a final note, Figure 22 illustrates the comparison of the surface appearances of PC used in this study, obtained under the Seosan outdoor weathering test and the indoor accelerated weathering test per SAE J2527 as shown in Table 7 [32]. Visible surface cracks appeared after 100 MJ/m^2^, and the density of the surface crack increased and multiplied as the UV exposure was increased. However, the visible surface crack did not appear with outdoor exposure even after receiving 1020 MJ/m^2^ UV irradiation. The surface cracking occurred when the load was applied during the tensile test. Therefore, it is clear that since not all weathering factors can be reproduced in accelerated laboratory tests [33], establishing the baseline dataset from the actual outdoor testing is essential for validating the accelerated test results.

## 4. Conclusions

An extensive 4-year outdoor weathering test on PC, PP, PBT, and HDPE polymers was carried out to provide basic long-term stress–strain data to predict the performance of plastic parts undergoing weathering deterioration. The total UV irradiation received was 1020 MJ/m^2^ at 315~400 nm wavelength, and they were tensile tested at every 60 MJ/m^2^ interval. The stress–strain data confirmed that the tensile modulus, tensile-yield strength, and yield strain for all four polymers did not change to a noticeable degree with prolonged weather exposure. The fracture strain, on the other hand, underwent a significant reduction. An inverted S-shape behavior described the fracture strain change. Initially, a small change was observed, then an accelerated decrease followed by converging to a minimum value, representing complete mechanical property degradation. For PP, PBT, HDPE, and PC, the % fracture strain retention was 47%, 27%, 20%, and 17%, respectively, over 4 years of outdoor exposure. With the identification of fracture strain retention as the key performance index for mechanical property degradation of weather-exposed polymers, a fracture strain retention function was developed using logistic regression analysis for each polymer. The fracture strain retention function was then applied to the true stress–strain curve, and the mechanical property degradation dataset was established for weathering FE analysis. An example FE analysis was carried out on the PC double chamber channel subject to impact loading at intermittent UV radiation doses. The results have shown that the fracture initiation and propagation energies upon impact loading were all affected by UV exposure. The fracture initiation energy was reduced as much as 90% when the fracture strain retention was 24%. In addition, fracture initiation energy was more affected than the propagation energy by about 20%. Thus, the potential of using fracture strain retention function combined with the true stress–strain data has been demonstrated to predict the performance of polymeric parts undergoing mechanical property degradation upon outdoor weathering. Finally, the importance of the outdoor weathering test in the validation of the accelerated laboratory testing was presented in terms of the surface crack formation, which can strongly affect the mechanical property degradation behavior of polymers.

## Figures and Tables

**Table 1:** Basic information on four polymers.

Material	UV Stabilizer	Tensile Modulus (MPa)	Tensile Yield Strength (MPa)	Fracture Strain (%)	MFR ^1^ (g/10 min)	Density (g/cc)	CTE ^2^, ×10^−5^ (mm/mm/°C)
PC	Yes	2340	60	150	15 (300 °C/1.2 kg)	1.2	6.80
PP	Yes	1200	19	30	25 (230 °C/2.16 kg)	1.03	4.50
PBT	Yes	2200	50	>50	15 (250 °C/5.0 kg)	1.33	8.00
HDPE	None	800	28	>600	2 (190 °C/2.16 kg)	0.952	15.00

^1^ MFR: Melt Flow Rate, ^2^ CTE: Coefficient of thermal expansion.

**Table 2:** Four-year monthly average black panel temperatures at solar angle of 37°, facing south.

Black Panel Temperature @ 37° Facing South (°C)		December	January	February	March	April	May	June	July	August	September	October	November
Highest temp. (*T_max_*)		40.9	35.1	38.6	47.2	50.8	54.1	54.0	62.3	64.9	57.5	55.3	48.2
Avg. temp.		3.7	2.5	4.0	8.9	14.6	20.0	24.4	27.1	28.9	25.1	19.0	10.4
Lowest temp. (*T_min_*)		−11.6	−13.3	−11.4	−9.6	−3.9	2.6	8.6	13.7	16.0	0.0	0.2	−5.8
ΔT		52.5	48.4	50.0	56.8	54.7	51.5	45.4	48.6	48.9	57.5	55.1	54.0
Seasonal temp.	*T_max_*	40.9			54.1			64.9			57.5		
	*T_min_*	−13.3			−9.6			8.6			−5.8		
Daily temp. cycle	*(T_max_ + T_min_)*/2	13.8			22.3			36.8			25.9		
	*(T_max_ − T_min_)*/2	27.1			31.9			28.2			31.7		
Seasonal temp. cycle	*(T_max_ + T_min_)*/2	25.8 = (64.9 + (−13.3))/2											
	*(T_max_ − T_min_)*/2	39.1 = (64.9 − (−13.3))/2											

**Table 3:** Relationship between UV radiation dose (*D*) at 315~400 nm and UV exposure time, *t*, in days.

*D* (MJ/m^2^)	0	60	180	300	420	540	660	780	900	1020
*t* (days)	0	128	246	461	579	794	913	1127	1246	1460

**Table 4:** Four-year mean values of mechanical properties of weather-exposed polymers.

Material	Tensile Modulus (MPa)		Tensile Yield Strength (MPa)		Strain at Yield (%)	
	Average	Standard Dev.	Average	Standard Dev.	Average	Standard Dev.
PC	2489.17	69.75	65.01	1.14	2.64	0.08
PP	1465.53	248.72	18.56	0.73	3.40	0.90
PBT	2409.32	108.27	54.04	1.52	4.09	0.32
HDPE	1105.20	96.11	27.55	0.67	3.96	0.41

**Table 5:** Regression parameters of each polymer for inverted S-shaped model for fracture strain variation.

$P_{e_{f}}\left(D\right)=\frac{e_{f_{D}}}{e_{f_{0}}}=P_{min}+\frac{P_{max}-P_{min}}{1\;+\;exp^{\left(k\left(D-D_{0}\right)\right)}}$		PC	PP	PBT	HDPE
Regression Parameters	*P_max_*	1.000	1.000	1.000	1.000
	*k*	7.3182 × 10^−^^3^	1.0493 × 10^−2^	1.5251 × 10^−2^	9.6216 × 10^−^^3^
	*P_mid_ = D*_0_	365.357	309.338	212.623	355.052
	*P_min_*	0.165	0.467	0.272	0.195
$R^{2}$		0.984	0.952	0.933	0.968
*D*_50%_ (MJ/m^2^)		420.199	567.934	264.246	406.344
$e_{f_{0}}$(%)		116.91	66.64	57.93	897.26

**Table 6:** Summary of impact analysis for weather-exposed PC channel section.

Cumulative UV Irradiation (MJ/m^2^ @ 315~400 nm)	Tup Displacement (mm) @ Max. R.F.	Max. R.F. (N)	Fracture Initiation Energy (J)	Fracture Propagation Energy (J)	Total Energy (J) @ 60 mm Tup Displacement	Reduction of Tup Displacement (%)	Reduction of Max. R.F. (%)	Reduction of Fracture Initiation Energy (%)	Reduction of Fracture Propagation Energy (%)	Reduction of Total Energy @ 60 mm Tup Displacement (%)
0	34.6	59,597.1	1391.26	916.78	2308.04	0.0	0.0	0.0	0.0	0.0
240	29.91	58,412.8	1157.57	808.32	1965.89	13.6	2.0	16.8	11.8	14.8
480	15.85	46,087.6	389.43	531.51	920.94	54.2	22.7	72.0	42.0	60.1
720	9.34	31,882.0	136.76	367.58	504.34	73.0	46.5	90.2	59.9	78.1
1020	8.52	27,809.2	111.87	264.89	376.76	75.4	53.3	92.0	71.1	83.7

**Table 7:** Accelerated weathering test cycles.

Step	Water Spray	Irradiance (W/m^2^ @ 340 nm)	Humidity (%)	Air Temperature (°C)	Black Panel Temperature (°C)	Duration (min)
1	Off	0.55	50	47	70	40
2	On	0.55	95	47	70	20
3	Off	0.55	50	47	70	60
4	On	0	95	38	38	60

## Data Availability

The data that supports the findings of this study are available within the article.
